# A liquid- and capsule-compatible hydroxyurea electronic monitor to identify non-adherent youth with sickle cell disease

**DOI:** 10.1371/journal.pone.0358450

**Published:** 2026-10-21

**Authors:** Anyssa Young, Nate Goldfarb, Patrick McGann, Sherif M. Badawy, Sebastian Seiguer, Savannah Winkler, Erika Huerta, Lisbel Gonzalez, Saige Armistead, Susan Creary

**Affiliations:** 1 Abigail Wexner Research Institute, Nationwide Children’s Hospital, Columbus, Ohio, United States of America; 2 Hasbro Children’s Hospital, Pediatric Hematology/Oncology, Lifespan Comprehensive Sickle Cell Center, Providence, Rhode Island, United States of America; 3 Ann & Robert H. Lurie Children’s Hospital of Chicago, Division of Hematology, Oncology, and Stem Cell Transplant, Chicago, Illinois, United States of America; 4 Department of Pediatrics, Northwestern University Feinberg School of Medicine, Chicago, Illinois, United States of America; 5 Scene Health, Brooklandville, Maryland, United States of America; 6 Brown University Health, Pediatric Hematology and Oncology, Providence, Rhode Island, United States of America; 7 Department of Hematology, Oncology, and Bone Marrow Transplant, Nationwide Children’s Hospital, Columbus, Ohio, United States of America; Imam Abdulrahman Bin Faisal University, SAUDI ARABIA

## Abstract

Prescription and claims data indicate that hydroxyurea (HU) adherence is low among youth with sickle cell disease (SCD). Electronic monitors (EM) are a gold standard measure, yet existing models have significant drawbacks that limit their use in youth with SCD. We developed a small, cellular-connected EM that is compatible with both liquid and capsule HU formulation bottles. In testing this EM among a sample of adolescents and young adults and caregivers of young children with SCD, we found that the EM is feasible and acceptable among youth with SCD and caregivers of young children with SCD in the short-term. We also found that the EM are useful for determining HU adherence and gained user insights that may improve their use longer-term. Future studies should examine the EM ability to determine which youth with SCD could most benefit from HU adherence intervention and treatment escalation in those with optimized adherence.

## Introduction

Sickle cell disease (SCD) is a hereditary hemoglobinopathy affecting approximately 100,000 Americans, with complications that can include acute vaso-occlusive pain and acute chest syndrome [1]. Hydroxyurea (HU) is the primary disease-modifying therapy used to treat SCD [2], however prescription and claims data show that HU non-adherence is a challenge facing approximately 50% of youth with SCD and that it has negative consequences on outcomes [3–7]. The ability to identify individuals who are non-adherent is critical so that adherence interventions and resources can be directed towards those who need them and have potential to benefit. Unfortunately, prescriptions, claims, hematologic parameters, and self-report data may not be practical and/or valid methods to identify the individuals who are non-adherent to HU in clinical practice [8].

Electronic monitors (EM) are a gold standard medication adherence measure that have the ability to identify non-adherence at the patient level [9,10]. However, existing EM have significant drawbacks, limiting their use among youth with SCD. For instance, MEMS^®^ bottles and GlowCap^™^ are not compatible with liquid medication formulations and must be returned by the user to abstract their adherence data [11–13]. This prevents the ability to use these EM among young children who are prescribed liquid HU and/or determine HU adherence in real-time. While Wise device EM (Wisepill and Wisebag) are compatible with the different HU formulations and include wireless technology to avoid having to return the devices, a study among youth with SCD suggests that Wisepill may be too small to hold a month supply of HU and that Wisebag may be too large to store in the refrigerator, which is required for liquid HU [14].

To address these gaps, our team developed a small, cellular-connected, liquid- and capsule-compatible EM. These EM have a medication holder, a button to click that has a green light that illuminates when clicked, and a strap that attaches it to the outside of any pharmacy-dispensed HU bottle (Fig 1). Users are instructed to attach the HU bottle to these devices and to self-report that they or their child took HU by clicking the button on the EM when HU is administered. EM adherence data from these button pushes are uploaded to a secure cloud-based dashboard. Understanding how these EM are received in youth with SCD is needed to inform design improvements and ensure that they are a reliable and valid method to identify youth who could benefit from adherence intervention. Thus, the aims of this study were to describe the feasibility and acceptability of these EM for 30 days and to describe HU adherence captured by these EM.

**Fig 1:**
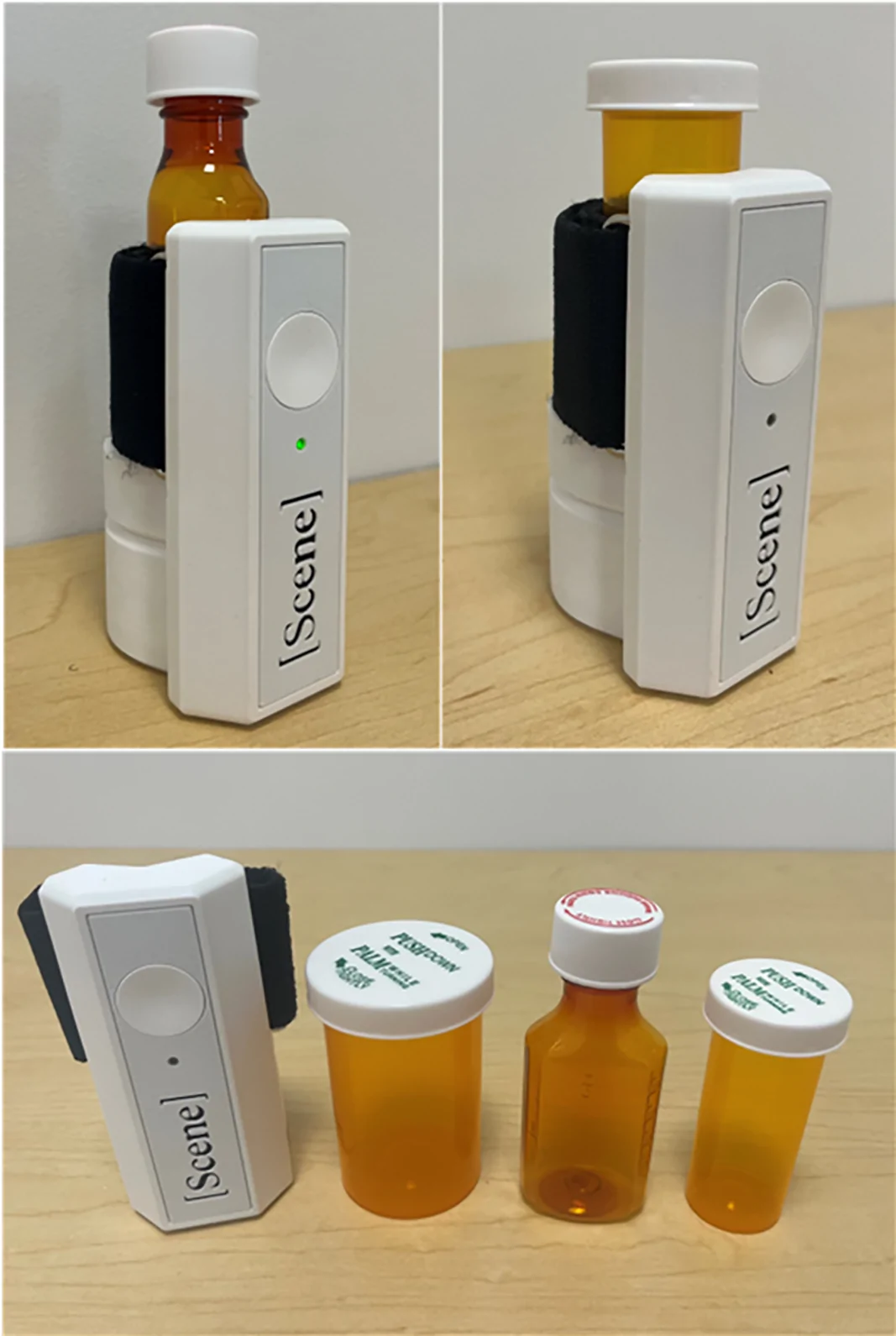
The EM device.

## Materials and methods

Participants were primary caregivers (CG) of children with SCD aged <11 years and adolescents and young adults (AYA) aged 11–25 years with SCD who enrolled in a HU adherence intervention randomized clinical trial (NCT: 06264700). Following approval from Nationwide Children’s Hospital’s Single Institutional Review Board, written consent was obtained from primary CG participants, CG of AYA aged ≤18 years, and AYA aged ≥18. Study recruitment of all participants lasted from 7/11/2024–7/10/2025. All subjects received the EM and were given instructions at enrollment to press the button on the device each day during the study period at the time that they took their HU or gave HU to their child.

Demographic data were self-reported by CG and abstracted from the electronic medical record for AYA. EM use and adherence data were abstracted from the secure dashboard for the 30 days after enrollment and prior to randomization. Since HU is prescribed as a once-daily medication, participants were only counted as adherent for days that they had at least one button push between 12:00AM to 11:59PM. Additional pushes that occurred during that 24 hour period were not considered when calculating the 30-day EM adherence rate.

Acceptability and the self-reported impact of the EM on HU adherence were assessed 30–120 days after enrollment to align with participants’ routine hematology care visits and when randomization to the adherence promoting-intervention or the control arm occurred [15]. This electronic monitor satisfaction survey included nine Likert scale items (Fig 2) and one open-ended response item. The open-ended item was analyzed using a qualitative description approach and representative responses were selected to exemplify participants’ perspectives on the EM [16]. This survey was a modified version of the survey used to evaluate electronic HU adherence interventions in previous studies [4,17].

**Fig 2:**
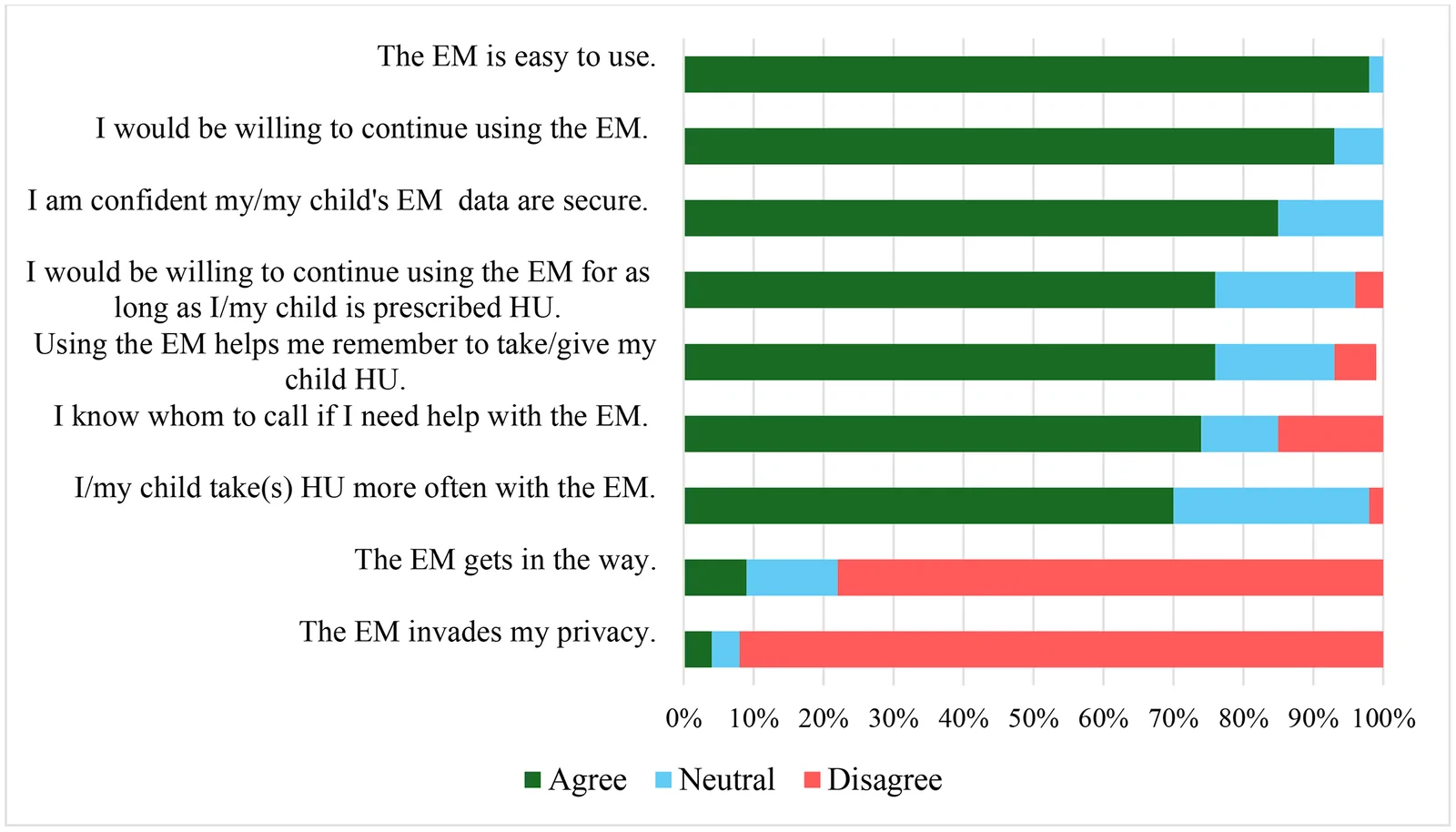
Adherence EM satisfaction survey results (n = 46).

Feasibility was defined as ≥80% of subjects using the EM ≥ 1 time in the first 30 days. For participants who used their device ≥1 time, we also collected their pattern of use (e.g., using after 14 days, using at least weekly). Acceptability was assessed using the satisfaction survey results. Adherence rates were calculated by dividing the number of days that the device was clicked at least once by 30. Participants (or their young children taking HU) were defined as adherent to HU if they used the EM for ≥24 days (≥80%) during the 30-days.

## Results and discussion

We enrolled 60 participants (Table 1). Of these, 46 (78%) completed the satisfaction survey (Fig 1). Of those enrolled, 54 (91% of AYA and 89% of CG) used the EM ≥ 1 time, 47 (78%) were using it after 14 days, and 32 (53%) used it at least weekly during the 30-day period. Among those who used the EM, the median HU adherence rate was 45% (IQR: 29–72%), and 11 (19%) were considered adherent to HU, including 7% of AYA and 36% of children of CG participants.

**Table 1:** Participant Demographics (n = 60).

Characteristic	AYA with SCD (n, %)	CG of child with SCD (n, %)
**Total Participants**	32 (53)	28 (47)
**Gender**		
Female	18 (56)	21 (75)
Male	13 (41)	7 (25)
Non-binary	1 (3)	–
**Race**		
Black/African American	23 (72)	23 (82)
African	4 (13)	3 (11)
Multiple Races	4 (13)	1 (4)
White	1 (3)	–
Not Listed	–	1 (4)
**SCD Genotype of youth with SCD**		
Hemoglobin (Hb) SS	25 (78)	23 (82)
Hb SC	5 (16)	3 (11)
Hb Sß^+^	1(3)	–
Hb Sß^0^	1 (3)	1 (4)
Other	–	1 (4)
**Age of youth with SCD (years)**		
0-5	–	10 (36)
6-10		18 (64)
11-17	21 (66)	–
18-25	11 (34)	–
**Length of time on HU**		
6 months to 1 year	1 (3)	5 (18)
1 year to 2 years	3 (9)	2 (7)
2 years or longer	28 (88)	21 (75)

Many participants reported qualitatively that they liked the EM and that it was easy to use, with statements like: “it’s fun and easy to use,” and “great as it is.” A few participants commented on the device size, stating, “The size should be adjusted,” and “it doesn’t fit the bottle but works well and I do like pushing the button.” Other participants remarked on the need for more instruction on how to use the EM, with one participant stating, “Explain a little more in detail about how to use the device and what the light will look like when it is ready or done.” Another participant shared that they desired more information on data security are and how effective these devices are stating, “Need to know how helpful it is supposed to be for parents or users. Are data secured for patients?” Lastly, multiple participants shared that they felt it helped them take their medication more often, with one participant stating, “I think the electronic adherence monitor is easy, simple to use. It’s helpful to remember to take my medication since it builds a routine for my medication,” and another shared, “I think it helps me keep track of taking his [my child’s] medication more often.”

Despite previous challenges using EM to quantify adherence for youth with SCD, our findings support that capturing a youth’s HU adherence with this EM is feasible, at least in the short term. This EM’s small size, ability to attach to any medication bottle, and its wireless capabilities are design improvements compared to other EM for HU that may have resulted in high acceptability ratings [11–14,18]. However, we did find that use declined over time, with participant use dropping from approximately 90% initially to 75% be week two. While this may reflect that users’ HU adherence declined, these data, coupled with our survey data, could also suggest that there are still opportunities to improve this EM if these devices are going to be used on a wider scale and longer-term to measure adherence intervention efficacy. For instance, survey and qualitative results, as well as previous literature on MEMS^®^ acceptability, suggest that ease of use, size, shape, and ability to discreetly use these devices are important to users [18], so modifications to make these devices more inconspicuous may need to be considered and trialed. It is also possible that future iterations may need to consider automating adherence measurement (e.g., include an electronic scale on the medication holder, rather than a button click, that detects when a dose of the medication was removed from the bottle).

It is notable that ~70% of participants reported that the EM helped to promote adherence and reminded them to administer HU. While recent literature suggests that EM alone may not improve adherence unless they are tied to other interventions [19], our findings align with older literature suggesting that EM may independently improve adherence in the short-term or temporarily [20,21]. While this study was limited by its small sample size, short duration, and the variability in the timing of survey administration, adherence data captured from these EM suggests that adherence supports beyond monitors are needed for many youth [4,9,10], especially AYA, with SCD [22,23].

## Conclusion

In summary, we identified that our EM was feasible and acceptable to use to identify non-adherent youth with SCD, likely because it addressed many prior EM challenges. However, there may need to be additional changes, specifically to make the EM less disruptive and so that AYA with SCD and caregivers of children with SCD are willing to use it longer-term. Future studies are warranted to validate the accuracy of these EM and their ability to delineate the youth who have potential to improve with adherence intervention. This is critical because the HU adherence strategies, like video directly observed therapy, smartphone apps, and community healthcare worker interventions, that have been tested only show a modest impact on outcomes. However, this may have been because adherent youth with SCD who had little potential to improve their outcomes from a HU adherence intervention were able to enroll in these studies [4,5,24,25]. Thus, future trials could consider using EM to identify who should be included to reduce sample sizes and cost and potentially improve trial success. Finally, in the era of expanding and expensive SCD treatments [26–28], another potential use for this EM could be to objectively determine when HU adherence is optimized and to inform which youth with SCD should consider treatment escalation.

## Supporting information

S1 File EMData. (XLSX)

S2 File DataSharing.9.23.26. (XLSX)
